# Proteomic Profiling of Cerebrospinal Fluid and Its Extracellular Vesicles from Extraventricular Drainage in Pediatric Pilocytic Astrocytoma, towards Precision Oncology

**DOI:** 10.3390/cancers16061223

**Published:** 2024-03-20

**Authors:** Sonia Spinelli, Xhuliana Kajana, Andrea Garbarino, Martina Bartolucci, Andrea Petretto, Marco Pavanello, Enrico Verrina, Giovanni Candiano, Isabella Panfoli, Maurizio Bruschi

**Affiliations:** 1 Laboratory of Molecular Nephrology, IRCCS Istituto Giannina Gaslini, 16147 Genoa, Italy; soniaspinelli@gaslini.org (S.S.);; 2 Proteomics and Clinical Metabolomics Unit at the Core Facilities of IRCCS Istituto Giannina Gaslini, 16147 Genoa, Italy; martinabartolucci@gaslini.org (M.B.);; 3 Department of Neurosurgery, IRCCS Istituto Giannina Gaslini, 16147 Genoa, Italy; 4 Unit of Nephrology and Kidney Transplant, IRCCS Istituto Giannina Gaslini, 16147 Genoa, Italy; enricoverrina@gaslini.org; 5 Department of Pharmacy, School of Medical and Pharmaceutical Sciences, University of Genoa, 16126 Genoa, Italy; 6 Department of Experimental Medicine (DIMES), University of Genoa, 16126 Genoa, Italy

**Keywords:** cerebrospinal fluid, extraventricular drainage, extracellular vesicles, small extracellular vesicles, large extracellular vesicles, proteomics, pilocytic astrocytoma

## Abstract

**Simple Summary:**

Childhood pilocytic astrocytoma (PA) is the most prevalent brain tumor in children, the pathogenesis of which remains incompletely understood. The identification of PA biomarkers is crucial for precise treatment and follow-up in affected patients. Cerebrospinal fluid (CSF), which is in close proximity to the brain and serves as a route for metastases, holds promise as a source for biomarker discovery. Our study entails a proteomic characterization of CSF collected from extraventricular drainage waste, as well as its extracellular vesicles, which express valuable disease targets. In a cohort of 19 PA patients, two proteins (inactive carboxypeptidase-like protein X2 and aquaporin-4) were found to be statistically significantly deregulated compared to controls of 18 children with congenital hydrocephalus (nontumoral controls) and 13 with medulloblastoma (unrelated tumoral controls). These proteins are considered promising potential biomarkers for validation in the blood through subsequent studies into the clinical application of minimally invasive translational screening.

**Abstract:**

Pediatric pilocytic astrocytoma (PA) is the most common brain tumor in children. Complete resection provides a favorable prognosis, except for unresectable PA forms. There is an incomplete understanding of the molecular and cellular pathogenesis of PA. Potential biomarkers for PA patients, especially the non-BRAF-mutated ones are needed. Cerebrospinal fluid (CSF) is a valuable source of brain tumor biomarkers. Extracellular vesicles (EVs), circulating in CSF, express valuable disease targets. These can be isolated from CSF from waste extraventricular drainage (EVD). We analyzed the proteome of EVD CSF from PA, congenital hydrocephalus (CH, non-tumor control), or medulloblastoma (MB, unrelated tumoral control) patients. A total of 3072 proteins were identified, 47.1%, 65.6%, and 86.2% of which were expressed in the unprocessed total and in its large-EV (LEV), and small-EV (SEV) fractions. Bioinformatics identified 50 statistically significant proteins in the comparison between PA and HC, and PA and MB patients, in the same fractions. Kinase enrichment analysis predicted five enriched kinases involved in signaling. Among these, only Cyclin-dependent kinase 2 (CDK2) kinase was overexpressed in PA samples. PLS-DA highlighted the inactive carboxypeptidase-like protein X2 (CPXM2) and aquaporin-4 (AQP4) as statistically significant in all the comparisons, with CPXM2 being overexpressed (validated by ELISA and Western blot) and AQP4 downregulated in PA. These proteins were considered the most promising potential biomarkers for discriminating among pilocytic astrocytoma and unrelated tumoral (MB) or non-tumoral conditions in all the fractions examined, and are proposed to be prospectively validated in the plasma for translational medicine applications.

## 1. Introduction

Childhood Central Nervous System (CNS) tumors represent 25% of pediatric tumors and are the leading causes of cancer-related mortality in children [1]. The most common are low-grade gliomas, categorized by the WHO into four grades: I (pilocytic astrocytoma, PA), II (diffuse astrocytoma), III (anaplastic astrocytoma), and IV (glioblastoma multiforme) [2]. PA is one of the most prevalent CNS tumors in the pediatric population, representing a complex medical condition [3]. PA can be sporadic or associated with type 1 neurofibromatosis (15%) and is characterized by its predominantly benign nature, slow growth, and favorable prognosis upon complete resection [4]. The recent review by Bauman et al. (2022) adds to our understanding of childhood PA [5]. Maximal resection is the recommended first-line treatment, as its extent significantly influences recurrence-free survival. Advances in surgical and adjuvant therapy have improved the 5-year survival (now around 75%) of children with low-grade glioma [6]. However, radical resection is not always feasible, particularly for deep midline supratentorial PAs whose resectability depends on the anatomical site. Although 32% of PAs occur mostly in the posterior fossa, they are also found in the cerebral cortex, optic pathways, hypothalamus, brainstem, and spinal cord [2]. About 90% of pediatric PAs exhibit genetic alterations in the RAS-mitogen-activated protein kinase (MAPK) pathway originating from activation of the v-Raf murine sarcoma viral oncogene homolog B (BRAF) [3]. B-Raf aberrant kinase is utilized for targeted treatment in the BRAF-mutated PA forms: inhibitors such as vemurafenib and dabrafenib have shown promise in preclinical studies and clinical trials on PA [7,8]. The study by Kim et al. (2023), comparing clinical features and treatment outcomes in pediatric and adult patients with PA, offers valuable insights into age-related differences [9]. However, some childhood PAs exhibit clinical variability, recurrence, and poor progression-free survival. Among these are the non-BRAF-mutated and those with increased mitotic activity and necrosis, called PA with anaplasia (PA-A), which are not amenable to immunotherapy [10]. The non-BRAF-mutated and PA-A lack a therapeutic target, being dependent on current chemotherapy, whose morbidities can influence the patient’s long-term quality of life [11]. Kinases are key regulators able to influence cellular processes such as carcinogenesis [12]. Protein phosphorylation controls biological processes [13]. Recent studies uncovered additional kinase mutations besides B-Raf, including Raf-1 proto-oncogene (RAF1), fibroblast growth factor receptor 1 (FGFR1), and others [14]. The expanding repertoire of kinases involved in childhood PA needs a comprehensive understanding. Liquid biopsy, evaluating circulating tumor DNA, RNAs, cells, and extracellular vesicles (EVs) by high-throughput technologies, can identify potential biomarkers and key signaling pathways [15]. A study [16] emphasized the potential of DNA methylation profiling as a powerful tool in distinguishing between various pediatric brain tumors, including PAs. However, liquid biopsy studies on low-grade gliomas have remained less conspicuous than those on other malignant brain tumors. The use of serum presents a major hindrance to the finding of brain-specific protein markers, due to the selectivity of the blood–brain barrier [17]. By contrast, cerebrospinal fluid (CSF) is a highly promising source for biomarker discovery, being accessible and in contact with both brain tissue and tumor bulk, and a primary route for metastases. Previous mass spectrometry studies have shown that CSF contains many unique proteins and has been considered a biochemical clinical window on the brain [18]. However ethical and volume limitations apply to CSF collection by lumbar puncture. By contrast, the use of CSF from extraventricular drainage (EVD) allows for large-volume, serial samplings [19]. The absence of volume restriction allows for the isolation of extracellular vesicles (EVs) from EVD CSF. Based on size, EVs can be classified as large EVs (LEVs) (>200 nm), and small EVs (SEVs) (<200 nm) [20]. EVs are released by all cell types and are present in biological fluids. EVs also transfer signals between cells at a distance and carry active molecules, as well as having a metabolic capacity [21,22]. EVs have garnered increasing attention as potential reservoirs of biomarkers in various cancers [23,24]. Numerous studies have shown that EVs play a leading role in the regulation of tumor microenvironment and tumor progression, through the transfer of proteins and genetic material [25]. A seminal study demonstrated the presence of tumor-specific RNA in circulating SEVs in glioblastoma patients [26]. Our previous proteomic study of EVD CSF and its LEVs and SEVs showed that it is possible to discriminate between childhood brain tumoral and nontumoral conditions, regardless of the kind of tumor [19]. Moreover, bioinformatic analysis of our previous data of EVD CSF and its EVs from medulloblastoma (MB) and matching controls showed that most of the potential disease biomarkers are found in the EVs and would be lost in the unprocessed total [27]. Comprehensive characterization of CSF-derived EVs from low-grade gliomas like PA is missing. Although diagnostic procedures are based on histopathology and neuroimaging, there is a need to identify novel PA biomarkers to facilitate therapeutic stratification and detection of residual disease and recurrence. The identification of reliable biomarkers can contribute to the development of non-invasive monitoring therapy response and disease progression, improving overall healthcare outcomes. The present study conducted a molecular characterization of CSF from EVD waste and the CSF-associated EVs as a source of new biomarkers for PA. Two kinds of controls were chosen, namely, children needing the insertion of an EVD for conditions unrelated to tumoral ones or for MB, a different tumoral disease. The paper also investigates the role of kinases in childhood PA.

## 2. Materials and Methods

### 2.1. Sample Size and Experimental Design

The selection of the optimal sample size is a critical parameter in the experimental design of biomarker discovery studies. The number of controls and patients were determined a priori, considering the biological variability of cerebrospinal fluid (CSF) extraventricular drainage (EVD) samples obtained in our previous studies [28]. The design included 80% of power and alpha error ≤ 0.05 after correction for multiple interactions (Benjamini–Hochberg) using the equations and procedures suggested by Dell RB et al. [29] and Forshed J [30]. Furthermore, to increase the probability of identifying new diagnostic biomarkers of pilocytic astrocytoma (PA), congenital hydrocephalus (CH), and medulloblastoma (MB) were used as unrelated to a brain tumor and as brain tumor unrelated to the low-grade glioma diseases, respectively.

### 2.2. Patients and Sample Collection

A total of 50 pediatric patients with CH with grades from III to V or with pilocytic PA or MB, admitted to the neurosurgery Unit of IRCCS Istituto Giannina Gaslini in the period between 2020 and 2023, who required the placement of an EVD catheter, were enrolled in the study. All patients with brain tumors had a histological diagnosis centrally reviewed and performed according to the World Health Organization (WHO) classification [31]. EVD CSF samples otherwise destined to waste were collected non-invasively at the first change of the disposable bag of EVD, by a sterile procedure. For this study, no seriate sampling was performed. Written informed parental consent was obtained before enrolment. In the discovery cohort, 24 randomly selected patients from CH (8), PA (8), and MB (8) were included, while a group of 50 EVD CSF samples including 18 CH, 19 PA, and 13 MB were included in the validation cohort of the study. The main demographic and clinical features of the subjects are summarized in Table 1. All collected EVD CSF samples were centrifuged at 3000× *g* for 10 min to remove cells and debris, aliquoted in different vials, and immediately frozen at −80 °C until use. All samples utilized for the discovery cohort were fractionated with three different biochemical–physical methodologies, obtaining the unprocessed total fraction (Tot) and its EV fraction including SEV and LEV fractions. Tot fraction was obtained from aliquots of 1 mL of CSF EVD, according to Bruschi et al. [27], while EVs were obtained from aliquots of 50 mL of CSF EVD by sequential ultracentrifugation, according to Bruschi et al. [27]. Briefly, after centrifugation at 3000× *g* for 10 min, the supernatant was centrifuged again at 7500× *g* for 15 min. The obtained supernatant was removed and centrifuged again at 22,000× *g* for 120 min to obtain the LEV faction. Finally, the supernatant was removed and centrifuged again at 100,000× *g* for 120 min to obtain the SEV fraction. All LEV and SEV pellets were rinsed in phosphate-buffered saline (PBS) and centrifuged again at 22,000× *g* for 1200 min for LEV or 100,000× *g* for 120 min for SEV samples. This rinse/centrifugation cycle was carried out five times in order to obtain clean LEV and SEV fractions. The size and homogeneity of the LEV and SEV fractions were checked by dynamic light scattering (DLS), using a Zetasizer nano ZS90 particle sizer (Malvern Instruments, Worcestershire, UK) at a 90° fixed angle in PBS at 25 °C, as reported by Bruschi et al. [32].

### 2.3. Mass Spectrometry Profile

Samples were lysed, reduced and alkylated in LYSE buffer (Preomics, Billerica, MA, USA), and the protein concentration was measured using a tryptophan assay [33]. Then 25 µg of each sample was digested by adding trypsin and LysC (at a 1:50 and 1:100 ratio of enzyme to sample protein, respectively, both in micrograms), mixing and incubating at 37 °C overnight. Digested samples were loaded onto StageTips [34]. The resulting peptides were completely dried using a speed vacuum centrifuge at 30 °C, suspended in 2% acetonitrile (ACN) and 0.1% formic acid (FA), and analyzed by a nano-UHPLC-MS/MS system using an Ultimate 3000 RSLC coupled to an Orbitrap Fusion Tribrid mass spectrometer (Thermo Scientific Instrument, Waltham, MA, USA).

For Tot fraction, elution was performed with an EASY spray column (75 μm × 50 cm, 2 μm particle size, Thermo Scientific) at a flow rate of 250 nL/min with a 150 min non-linear gradient consisting of an 8 min wash with 2% buffer B (80% ACN, 20% H_2_O, 5% dimethyl sulfoxide (DMSO) and 0.1% FA), then increasing to 30% B over 97 min, with a further increase to 50% B after 20 min, followed by a 5 min wash at 80% B and a 20 min re-equilibration at 2% B. Mass spectrometry (MS) scans were acquired at a resolution of 120,000 between 375 and 1500 *m*/*z* and an AGC target of 4.0E5. MS/MS spectra were acquired in the linear ion trap (rapid scan mode) after collision-induced dissociation (CID) fragmentation at a collision energy of 35% and an AGC target of 4.0 × 10^3^ for up to 250 ms. For precursor selection, the least abundant signals were prioritized. Ions with 2 *m*/*z* were scheduled for CID/IT analysis, with the same parameters applied as above. Charge states 3–7 with minimum precursor intensity of 500,000 were scheduled for analysis by a fast HCD/FT scan of maximal 40 ms at a resolution of 15,000. The remaining charge states of 3–7 ions with a maximum intensity of 500,000 were scheduled for analysis by CID/IT, as described above. Dynamic exclusion was set at 30 s.

For SEV and LEV fractions, elution was performed with an EASY spray column (75 μm × 50 cm, 2 μm particle size, Thermo Scientific) at a flow rate of 250 nl/min with a 100 min non-linear gradient consisting of a 3 min wash with 2% buffer B, then increasing to 6% after 4 min and then further increasing to 30% after 58 min and to 45% after 13 min, followed by a 2-min wash at 80% B and a 20 min re-equilibration at 2% B. MS1 and MS/MS spectra were acquired as described above, with the difference that charge states 3–5 with minimum precursor intensity of 500,000 were scheduled for analysis by a fast HCD/FT scan of maximal 40 ms at a resolution of 15,000 and the remaining charge states of 2–5 ions with maximum intensity of 500,000 were scheduled for analysis by CID/IT, as described above. Dynamic exclusion was set at 25 s.

MaxQuant software version 2.4.10.0 [35] was used to process data. A false discovery rate was set at 0.01 for the identification of proteins, peptides, and peptide–spectrum match (PSM). A minimum of 6 amino acids were required for peptide identification. The Andromeda engine, incorporated into MaxQuant software, was used to search the MS/MS spectra against the Uniprot human database (release UP000005640_9606 April 2018). In the processing, the variable modifications are Acetyl (Protein N-Term), Oxidation (M), and Deamidation (NQ); on the other hand, the Carbamidomethyl (C) was selected as a fixed modification.

### 2.4. Western Blotting

Antibody specificity for CPXM2 was evaluated by Western blot analysis, as previously described [27]. Briefly, aliquots of 20 µg of a pooled Tot fraction obtained from all HC, PA, and MB patients or biopsy tissue obtained from PA patients were dissolved in Laemmli buffer, i.e., 2% *w*/*v* SDS, 10% glycerol, and 62.5 mM Tris-HCl pH 6.8, separated by sodium dodecyl sulfate polyacrylamide gel electrophoresis, and then transferred to a nitrocellulose membrane for 30 min at room temperature (RT) using Trans-Blot SD cell (Bio-Rad, Hercules, CA, USA). Specifically, the biopsy tissue was homogenized with IKA T10 homogenizers with ice-cold Laemmli buffer plus protease cocktail inhibitors, centrifuged, and the supernatant collected, fractionated and frozen at −80 °C until use. The full-length membrane was blocked, rinsed, labeled, and detected with rabbit polyclonal anti-human CPXM2 antibodies (Sigma-Aldrich, St. Louis, MO, USA) diluted 1:1000 in 3% (*w*/*v*) bovine serum albumin (BSA) in PBS containing 0.05% *v*/*v* Tween-20 (PBS-T). After rinsing in PBS-T, the membrane was incubated with HRP-conjugated secondary antibody diluted 1:10,000 in 1% *w*/*v* BSA in PBS-T. The chemiluminescence signal was acquired and quantified using the ChemiDoc and Quantity One software 4.6.8 (Bio-Rad, Hercules, CA, USA), respectively. Gel electrophoresis of the same samples was used as loading control and the image was digitized by GS-800 Densitometer (Bio-Rad).

### 2.5. ELISA Assay

CPXM2 was quantified in the validation cohort including 18 CH, 19 PA, and 13 MB samples using a direct homemade ELISA assay. Briefly, the Nunc MaxiSorp™ ELISA plate (Thermo Fisher) was coated with 5 µg of Tot fraction sample diluted in carbonate buffer and incubated overnight at 4 °C. Uncoated wells were used for background subtraction. Samples were removed and the wells were blocked with 3% BSA in PBS overnight at 4 °C. The wells were washed three times in PBS containing 0.05% *v*/*v* of Tween 20 (PBS-T), followed by incubation with 100 µL of polyclonal rabbit anti-human CPXM2 (Sigma-Aldrich) diluted 1:1000 in PBS-T containing 3% *w*/*v* BSA overnight at 4 °C. The wells were washed again three times with PBS-T and incubated with secondary antibody HRP-conjugated diluted 1:2000 in PBS-T containing 1% *w*/*v* BSA for 1 h at RT. Finally, the wells were washed again three times with PBS-T and developed with 3,3′,5,5′-tetramethylbenzidine substrate (Bio-Rad). The reaction was quenched by adding spot solution and the absorbance was measured at 450 nm using an iMark plate reader (Bio-Rad). The CPXM2 titers were determined using a standard curve derived from serial two-fold dilutions of a polled PA patient’s sample, whose titer was arbitrarily defined as 1 relative unit for ml (RU/mL). These reference standards were run in triplicate. A box plot was used to visualize the protein intensity. The lower detection limit was determined as the lowest protein intensity that could be differentiated from a blank.

### 2.6. Statistical Analysis

Statistical analysis was performed as previously described [28], with some modifications. Briefly, after log2 conversion, identified proteins were filtered for 70% presence in at least one fraction. Then, missing values were imputed using a normal distribution, and the whole dataset was normalized using the quantile method and analyzed by unsupervised hierarchical clustering (multidimensional scaling with k-means) and Spearman’s correlation, to identify outliers and dissimilarity between samples. The ANOVA test for unpaired samples was used to identify the proteins that changed statistically. Then, to identify the proteins that maximized the discrimination between each fraction in each clinical group, and between all PA and non-PA samples, we applied the *t*-test. For the ANOVA and *t*-test, proteins were significantly differentially expressed between two conditions with a power of 80% and an adjusted *p*-value ≤ 0.05 after correction for multiple interactions (Benjamini–Hochberg). Volcano plots were used to visualize the *t*-test analyses and the cutoff line was established using the function y = |c/(x −  x0)|. In addition, partial least squares discriminant analysis (PLS-DA) with the variable importance in projection (VIP) score was used to identify a priority list of importance of statistically significant proteins in the discrimination between PA and non-PA samples.

Gene set enrichment analysis was carried out to build a functional protein network based on their Gene Ontology (GO) annotation terms extracted from the Gene Ontology Consortium (http://www.geneontology.org/, accessed on 17 January 2024). After loading the protein expression data of the whole dataset, a ranked list was assigned to each GO annotation term. These ranks consider the number of proteins associated with each gene annotation term concerning all proteins identified, their mean fold change, and the *p*-value after correction with the false discovery rate (FDR) for multiple interactions. These rank values lie between −1 and 1, corresponding to minimal and maximal enrichment in each group fraction. In the two-dimensional scatter plot, the points that belong to the line passing between the points with coordinates (1x, 1y) and (−1x, −1y) represent the equally enriched GO annotation terms. The distance from this straight line is proportional to the increase in the annotation term enrichment in one of the two groups. Finally, kinase enrichment analysis (KEA) was also performed for statistically significant proteins, to identify the enriched kinases involved in the phosphorylation signaling of the discrimination of each fraction [36]. For the ELISA assay, the Kruskal–Wallis test was used to assess the difference in the intensity of the CPXM2 protein in the Tot fraction of the three different clinical groups. Results were expressed as medians and interquartile range (IQr). A receiver operating characteristic (ROC) curve was generated to assess the diagnostic efficiency of the ELISA assay in the discrimination between the PA and other patients. The AUC value was classified as the following: 0.5, not discriminant; 0.5–0.6, fail; 0.6–0.7, poor; 0.7–0.8, fair; 0.8–0.9, good, and 0.9–1, excellent. Youden’s index [37] and the likelihood ratio were used to identify the cutoff and the diagnostic performance of the ELISA assay, respectively. Two-sided *p*-values ≤ 0.05 were considered as significant. All statistical tests were performed using Origin Lab Pro 9.9 and the latest version of the software package R 4.3.3 available at the time of the experiments [38].

## 3. Results

### 3.1. Characterization of Extracellular Vesicles

The size and homogeneity of the LEV and SEV fractions obtained from the CSF EVD samples were confirmed by DLS, revealing a Gaussian distribution profile with a typical mean peak of LEVs (1000 ± 70) or SEVs (100 ± 5 nm), respectively (Figure S1) [20].

### 3.2. Protein Profile

**Figure 1:**
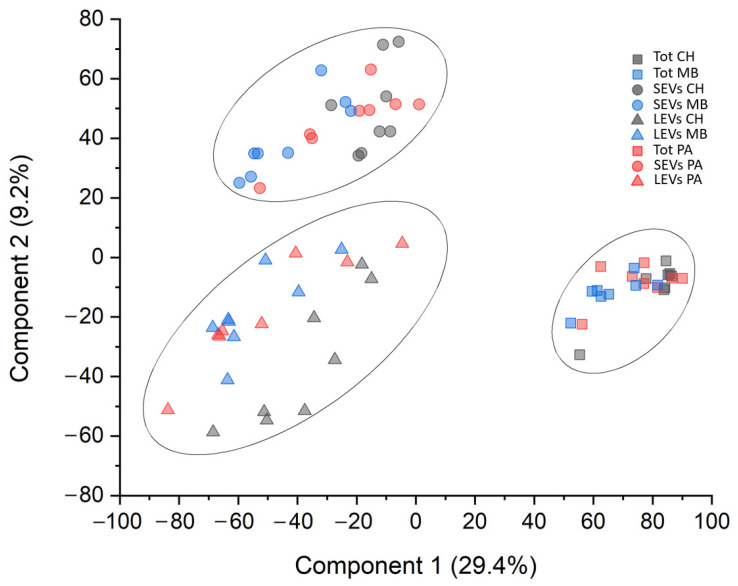
Multidimensional scaling with k-means of whole proteomic dataset of EVD CSF samples. Two-dimensional scatter plot of unsupervised cluster analysis of whole-protein dataset. The plot shows the absence of any outlier and the presence of three distinct clusters corresponding to unprocessed-total (square), small-EV (circle), and large-EV (triangle) fractions. On the other hand, there was no discrimination between the congenital hydrocephalus (CH) (gray), pilocytic astrocytoma (PA) (red), and medulloblastoma (MB) (blue) clinical groups.

A total of 3072 proteins were identified (Supplementary Table S1). Among these 3072 proteins, 982 (32%) of them were present in all fractions. Only 269 (8.7%), 109 (3.5%), and 548 (17.8%) proteins were exclusive to the Tot, SEV, and LEV fractions, respectively (Figure S2a). Furthermore, out of 1447 proteins identified in the Tot fraction, 1194 (82.5%), 1051 (72.6%), and 1100 (76%) were expressed in CH, PA, and MB patients, respectively. Among these 1447 proteins, 806 (55.7%) of them were present in all fractions. Only 155 (10.7%), 62 (4.3%), and 138 (9.5%) proteins were exclusive to the HC, PA, and MB samples, respectively (Figure S2b), whereas, out of 2105 proteins identified in the SEV fraction, 1224 (58.1%), 1111 (52.8%), and 1886 (89.6%) were expressed in HC, PA, and MB patients, respectively. Among these 2105 proteins, 829 (39.4%) of them were present in all fractions. Only 138 (6.5%), 53 (2.5%), and 627 (29.8%) proteins were exclusive to the CH, PA, and MB samples, respectively (Figure S2c), whereas, out of 2648 proteins identified in the LEV fraction, 1895 (71.6%), 2231 (84.2%), and 2164 (81.7%) were expressed in CH, PA, and MB patients, respectively. Among these 2648 proteins, 1538 (58.1%) of them were present in all fractions. Only 168 (6.3%), 199 (7.5%), and 177 (6,7%) proteins were exclusive to the CH, PA, and MB samples, respectively (Figure S2d). Out of 3072 identified proteins, filtered for 70% of identity in at least one condition, 39 (1.3%), 115 (3.7%), and 215 (7%) proteins were kinases, cluster differentiation molecules, and MEROPS, respectively. Despite a considerable overlapping of protein identity between the three fractions, multidimensional scaling (MDS) analysis evidenced clear discrimination of three clusters corresponding to Tot, Ev, and LEV fractions. On the other hand, there was no discrimination between the three different clinical groups (Figure 1).

**Figure 2:**
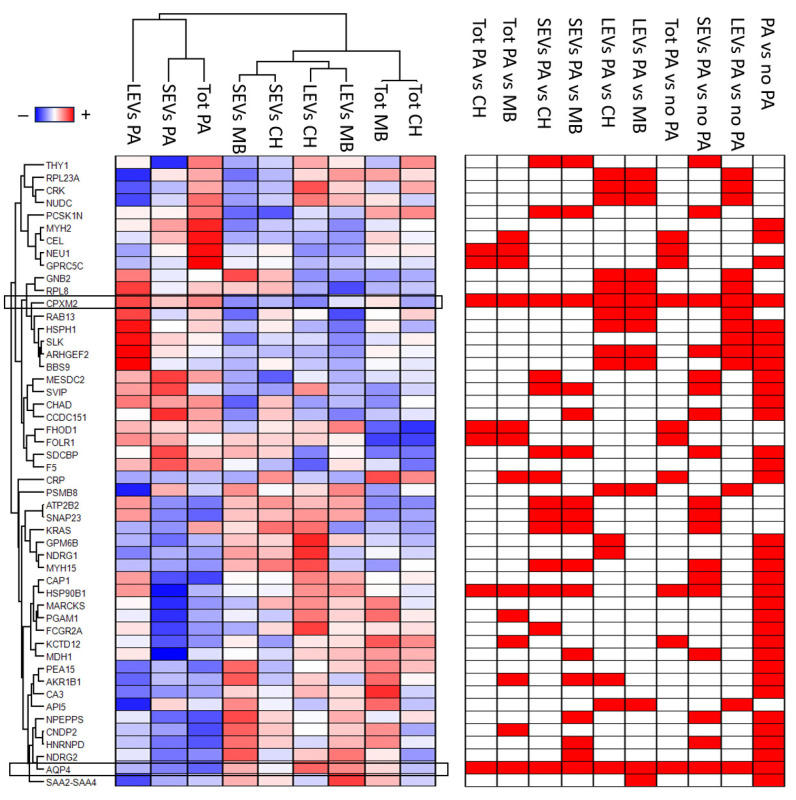
Heatmap of highly discriminant proteins for the different samples. Heatmap of 50 proteins highlighted by statistical analysis. In the heatmap, each row represents a protein, and each column corresponds to a condition. Normalized Z-scores of protein abundance are depicted by a pseudo color scale, with red indicating positive expression, white indicting equal expression, and blue indicating negative expression compared to each protein value, whereas the dendrogram displays the outcome of unsupervised hierarchical clustering analysis, placing similar protein profile values near each other. In addition, the diagram on the right of the heatmap reports the statistical significance (red) of each protein in each comparison. Visual inspection of the dendrogram and heatmap demonstrates the ability of these proteins to distinguish between pilocytic astrocytoma (PA), congenital hydrocephalus (CH), and medulloblastoma (MB) in unprocessed-total (Tot), small-EV (SEV), and large-EV (LEV) fractions (see detail in Supplementary Table S1). Carboxypeptidase-like protein X2 (CPXM2) was statistically more abundant in all PA samples compared to all other samples, whereas aquaporin-4 (AQP4) showed the opposite protein profile.

An ANOVA test for unpaired samples was used to identify the proteins that statistically distinguish between the three fractions in the three clinical groups. A total of 392 proteins were identified (Table S2). The expression profile of these proteins, after Z-score normalization, was visualized in the heatmap shown in Figure S3. Notably, 136 of these overlap with proteins identified as promising biomarkers of brain tumors in our previous work [28] (Table S2). Next, a T-test was applied to identify the proteins that distinguish between PA and HC, or PA and MB, or PA and non-PA patients for each fraction. Out of 1447 proteins identified in the Tot fraction, 13, 50, and 28 were statistically significant in the comparison of the PA vs. HC (Figure S4a), PA vs. MB (Figure S4b), and PA vs. non-PA (Figure S5a) samples, respectively (Table S2). Among these 62 statistically significant proteins identified in the Tot fraction, 7 (11.3%) were common to the three comparisons. Only 4 (6.4%), 30 (48%), and 6 (9.7%) proteins were exclusively statistically significant in the comparison between PA and HC, PA and MB, and PA and non-PA patients, respectively (Figure S6a). Out of 2105 proteins identified in the SEV fraction, 83, 67, and 39 were statistically significant in the comparison of the PA vs. HC (Figure S4c), PA vs. MB (Figure S4d), and PA vs. non-PA (Figure S5b) samples, respectively (Table S2). Among these 143 statistically significant proteins identified in the SEV fraction, 11 (7.7%) were common to the three comparisons. Only 57 (39.9%), 47 (32.9%), and 4 (2.8%) proteins were exclusively statistically significant in the comparison between PA and HC, PA and MB, and PA and non-PA patients, respectively (Figure S6b). Out of 2648 proteins identified in the SEV fraction, 181, 42, and 39 were statistically significant in the comparison of the PA vs. HC (Figure S4e), PA vs. MB (Figure S4f), and PA vs. non-PA (Figure S5c) samples, respectively (Table S2). Among these 217 statistically significant proteins identified in the SEV fraction, 13 (6%) were common to the three comparisons. Only 153 (70.5%), 25 (11.5%), and 7 (3.2%) proteins were exclusively statistically significant in the comparison between PA and HC, PA and MB, and PA and non-PA patients, respectively (Table S2 and Figure S6c). In addition, in order to identify the proteins that distinguish between PA and non-PA, regardless of the CSF EVD fraction, a *t*-test was also applied to compare PA and non-PA patients, considering all the three fractions together (Figure S5d). A total of 34 proteins were identified (Table S2). To obtain a visual illustration of differential expression proteins between each comparison performed, a volcano plot was produced (Figures S4 and S5). Notably, only 50 proteins were statistically significant in the comparison between PA and HC, and between PA and MB patients in the same CSF EVD fractions (Table S3). The expression profile of these 50 proteins, after Z-score normalization, is visualized in the heatmap shown in Figure 2. Furthermore, among these 50 proteins, only inactive carboxypeptidase like protein X2 (CPXM2) and aquaporin-4 (AQP4) were statistically significant in all the comparisons. The CPXM2 protein was overexpressed in PA patients in all the comparisons, whereas AQP4 showed the opposite protein profile (Figure 2, Figures S4 and S5). Notably, of these 50 highlighted proteins, 12 (24%) overlap with core panel proteins identified as the most promising biomarkers of brain tumors in our previous work, including AQP4 [28] (Table S2).

**Figure 3:**
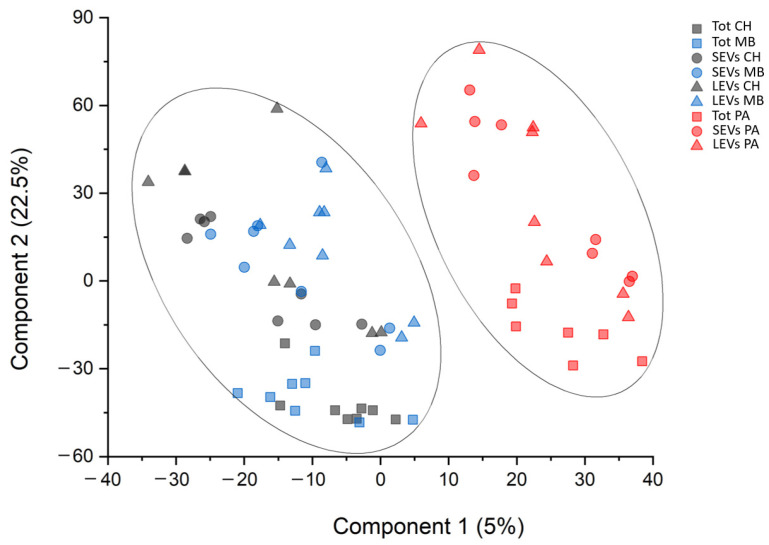
Partial least squares discriminant analysis (PLS-DA) of proteomic dataset. Two-dimensional scatter plot of PLS-DA score of CSF EVD protein-profile label-free quantitation intensity from pilocytic astrocytoma (PA) (red), congenital hydrocephalus (CH) (gray), and medulloblastoma (MB) (blue) in unprocessed-total (square), small-EV (circle), and large-EV (triangle) samples. Ellipses represent the 95% confidence intervals. The plot shows a clear separation between PA and non-PA samples.

Finally, PLS-DA analysis was performed in order to define a protein list for priority in the discrimination between PA and non-PA samples. This analysis identified a ranked panel of 392 statistically significant proteins (Table S2). Their priority was determined using the variable importance in the projection (VIP) score. Also, this analysis identified the CPXM2 and AQP4 proteins as proteins that maximize the discrimination between PA and non-PA patients, regardless of the CSF EVD fractions (highest VIP score). In addition, the k-means analysis associated with PLS-DA showed the presence of two distinct clusters, corresponding to PA and non-PA patients (Figure 3). This result indicated that PLS-DA was a good model for discrimination and prediction of PA and non-PA samples.

In summary, a total of 392 statistically significant proteins were identified, of which 50 were statistically significant in the comparison between PA and HC, and between PA and MB patients in the same CSF EVD fractions, and only the CPXM2 and AQP4 proteins were statistically significant in all the comparisons. These two proteins were therefore the most promising potential biomarkers in the discrimination of PA patients among the CH and MB patients, regardless of the CSF EVD fraction used.

### 3.3. Gene Ontology Enrichment Analysis

The considerable diversity in the expression profile of all the proteins identified in all conditions may imply their different biological roles. To assess this, we performed a Gene Ontology (GO) enrichment analysis based on the annotation terms extracted from the Gene Ontology Consortium (http://www.geneontology.org/, accessed on 17 January 2024). This analysis identified a total of 60 significantly enriched GO annotation terms in the comparison of PA and non-PA samples. Among these, 34 and 19 were enriched above or below the 95% of CI, respectively (see detail in Table S4). Results are visualized by a scatter plot (Figure S7), where each point corresponds to a GO annotation term. The points above or below the line with equation x = y were, respectively, enriched in PA or non-PA samples. These results could be summarized in the upregulation of proteins associated with glioma and, in particular, of the low-grade glioma, kinases, MEROPS and cell cycle proteins in the PA samples, while the proteins associated with the immunity and endopeptidase inhibitor activity were downregulated. Notably, in each CSF EVD fraction, the GO analysis shows the enrichment of the same, or highly similar, GO annotation terms in the PA samples.

Next, kinase enrichment analysis (KEA) [36] was conducted using as substrates all 392 statistically significant proteins identified to highlight the kinases most probably involved in the regulation of phosphorylation signaling in the PA samples. Out of 39 kinases identified, 6 (WNK1, CDK2, OXSR1, PTK7, and SLK), 2 (OXSR1 and PTK7), 3 (WNK1, CDK2, and OXSR1), 2 (CDK2 and SLK), and 1 (SLK) were statistically significant for the ANOVA test and in the comparison between PA vs. HC patients in the SEV fraction, PA vs. HC patients in the LEV fraction, PA vs. non-PA patients in the LEV fraction, and PA vs. non-PA patients considering the three different fractions together, respectively (Table S2). The expression profile of these kinases and their *p*-value are visualized in the bubble diagrams shown in Figure S8. Then, gene manes of all statistically significant proteins identified in this study with their expression profile and *p*-value were used for KEA analysis. This analysis predicted a total of five kinases statistically enriched (EGFR, CDK2, CSNK2A1, NTRK1 and LRR2, see detail in Table S5). Among these, only CDK2 and CSNK2A1 were identified in our experiment, but only CDK2 was statistically overexpressed in PA samples in at least one comparison (Table S1 and Figure S8). 

### 3.4. Western Blot Analysis

Primarily, the specificity of the rabbit polyclonal anti-human CPXM2 antibody was tested with Western blot analysis. As shown in Figure S9, the antibody detected a single band corresponding to the molecular weight of the entire protein. Furthermore, the band intensity confirms the results obtained in the MS-based approach. Blue-silver staining of the same samples was used as in the loading control of the Western blot analysis [39]. Moreover, the anti-human CPXM2 antibody was also tested in three paired samples of CSF EVD and biopsy tissue obtained from patients with pilocytic astrocytoma. As shown in Figure S10, the antibody detects a single band corresponding to the expected molecular weight of the entire protein.

### 3.5. ELISA Assays

**Figure 4:**
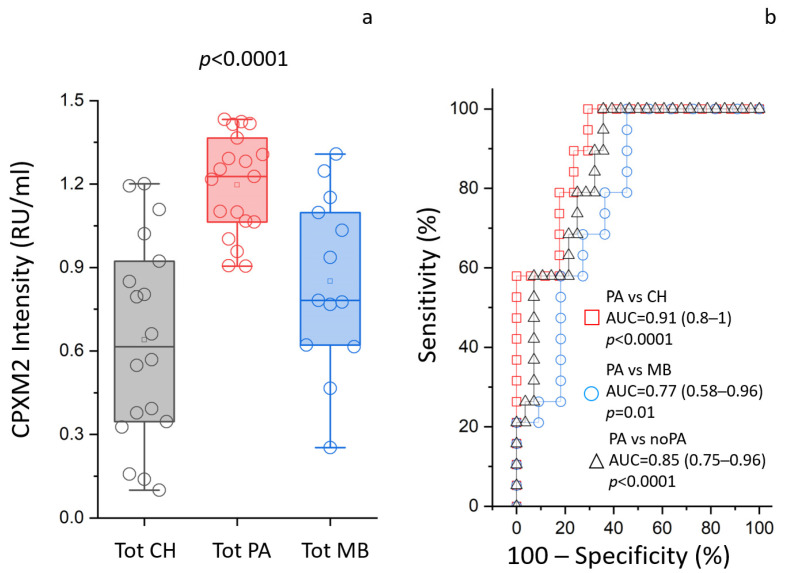
Inactive carboxypeptidase-like protein X2 (CPXM2) ELISA validation. (**a**) Box plots of direct homemade ELISA assay for CPXM2 protein in unprocessed total fraction (Tot) of EVD CSF obtained from congenital hydrocephalus (CH), pilocytic astrocytoma (PA), and medulloblastoma (MB) patients. CPXM2 is statistically more abundant in PA (*p* < 0.0001) samples compared to CH and MB samples. (**b**) ROC curve analysis for the CPXM2 ELISA assay.

A direct homemade ELISA assay was used to validate the potential role of a biomarker of CPXM2 expression in the Tot fraction obtained from the CSF EVD of HC, PA, and MB patients. Figure 4 shows that CPXM2 was statistically more abundant (*p* < 0.0001) in the Tot fraction of PA patients compared to the same fraction of CH and MB patients, with values of 1.38 (1.35–1.4), 1.23 (1.15–1.31), and 1.26 (1.22–1.36) relative units per milliliter (RU/mL), respectively. The comparison between PA and HC patients displayed an area under the curve (AUC), confidence interval (CI), and *p*-value of 0.91, 0.8–1, and <0.0001, respectively. The assay’s cutoff value, sensitivity, specificity, and likelihood ratio were found to be >1.29, 100%, 71%, and 3.4, respectively, while the comparison between PA and MB patients displayed an area under the curve (AUC), confidence interval (CI), and *p*-value of 0.77, 0.58–0.96, and 0.01, respectively. The assay’s cutoff value, sensitivity, specificity, and likelihood ratio were found to be >1.29, 100%, 54%, and 2.2, respectively, while the comparison between PA and non-PA patients displayed an area under the curve (AUC), confidence interval (CI), and *p*-value of 0.85, 0.75–0.96, and <0.0001, respectively. The assay’s cutoff value, sensitivity, specificity, and likelihood ratio were found to be >1.29, 100%, 64%, and 2.8, respectively.

## 4. Discussion

PA is the most frequent primary child brain tumor [4]. Despite its generally favorable outcome, the management of childhood PA needs an understanding of the tumoral molecular landscape for more effective follow-up strategies and treatment guidance [5]. Survival itself poses the problem of minimizing the morbidities of treatments in children. This study is a molecular profiling and a bioinformatic study of the proteome of EVD CSF of children bearing PA and of its LEVs and SEVs versus two kinds of controls, needing EVD insertion for HC or MB. The latter control was taken as an example of unrelated tumor. MB was shown to disseminate to the brain through the CSF [40]. MB is a malignant pediatric CNS tumor originating in the cerebellum from embryonic brain cells [41], classified by the World Health Organization as a grade IV tumor, in four distinct variants [31]. Proteomic analysis identified 3072 proteins in the CSF from the PA and controls. There was an overlapping of protein identity between the unprocessed-total (Tot), SEV, and LEV CSF fractions, but MDS analysis evidenced three clearly discriminated clusters corresponding to the three fractions. Indeed, notwithstanding the good discrimination between the Tot, SEV and LEV fractions, there was no discrimination between the three clinical groups. The role of EVs in intercellular communication and the transfer of bioactive molecules positions them as promising candidates for biomarker discovery in childhood PA. Recent proteomic data from the control EVD CSF showed that EVs are more informative than Tot CSF, as they express specific brain disease markers and therapeutic targets that cannot be detected in the Tot CSF, due to its considerable dynamic range, whereas the enriched EV fractions display a markedly lower variability [28]. Consistently, here 1447 (47.1%), 2105 (65.6%), and 2648 (86.2%) proteins were found expressed in the total, SEV, and LEV fractions, respectively. Among the 392 proteins defined by bioinformatic analyses as statistically significant in discriminating between PA and non-PA samples, 50 were statistically significant in the comparison between PA and HC and between PA and MB patients in the same CSF fractions. Some of the up-regulated proteins are known to be involved in cancer, such as retinoic acid-inducible G-protein coupled receptor (GPRC5C) [42] and Rho guanine nucleotide exchange factor (ARHGEF2), involved in cell cycle regulation [43]; Myosin-2 (MYH2), involved in cytoskeleton organization [44]; and the scaffold protein Syntenin-1 (SDCBP), typical of fetal life, whose gain-of-function has been associated with poor tumor prognosis [45]. There were also two chaperones, LRP (MESD), a modulator of the Wingless/Integrated (Wnt) pathway, and the heat shock protein 105 kDa (HSPH1), a nucleotide-exchange factor for chaperones HSPA-1A and -1B; this was consistent with our previous finding that HSP90 alpha can discriminate MB from all other brain cancers, in a proteomic analysis of EVD CSF from children bearing various CNS tumors [19]. HSP90 was also found overexpressed in MB [27]. Among the down-regulated proteins were heterogeneous nuclear ribonucleoprotein D0 (HNRNPD), which binds to RNAs of many proto-oncogenes [46]; BTB/POZ domain-containing protein (KCTD12), an auxiliary subunit of GABA-B receptors that increases agonist potency of the G-protein signaling of the receptors [47]; and, interestingly, some metabolic cytosolic enzymes (cytoplasmic malate dehydrogenase (MDH1), phosphoglycerate mutase (PGAM1), and the NADPH-dependent reductase Aldo-keto reductase family 1 member B1 (AKR1B1). 

The k-means analysis associated with PLS-DA showed that the analysis was able to discriminate PA from non-PA patients, forming two distinct clusters. PLS-DA highlighted CPXM2 (upregulated in PA) and AQP4 (downregulated in PA) as proteins that maximize the discrimination between PA and non-PA patients, regardless of the CSF fraction considered. As both AQP4 and CPXM2 were highlighted in all the comparisons, the study focused on these two proteins, which have the highest likelihood of being useful in identifying PA in any sample. Furthermore, also in our previous work, AQP4 has been identified as one of the most promising biomarkers of brain tumors [28]. The observation was validated by ELISA and Western blot analysis, limiting the analyses to the upregulated CPXM2. The ROC curve analysis indicated that CPXM2 has good diagnostic value as a biomarker for PA, not only in distinguishing the PA condition from nontumoral controls (CH), with AUC = 0.91, but also from another kind of tumor (MB), with AUC = 0.85. The relationship between AQP4 and brain tumor characteristics, such as invasion and migration, has been established. Notably, it was shown that inhibition of AQP4 mitigates the progression of brain tumors [48]. CPXM2 involvement in tumorigenesis or progression is less consistent. Recent studies on human osteosarcoma and gastric cancer reported CPXM2 overexpression as associated with an unfavorable prognosis [49]. Knockdown of CPXM2 in cultured osteosarcoma and gastric cancer cells hindered cell proliferation and migration. The knowledge emerged that CPXM2 acts in promoting epithelial to mesenchymal transition (EMT) [50]. Over the past decade, research has identified several kinases playing pivotal roles in the pathogenesis and progression of childhood PA [51]. The significance of MAPK pathway dysregulation, specifically the activation of BRAF gene fusions, primarily through KIAA1549-BRAF, was highlighted in most childhood PA [11]. This finding has broad implications for targeted therapeutic interventions, as evidenced by the promising results of clinical trials investigating BRAF inhibitors [51]. Here, KEA analysis conducted on the 392 statistically significant proteins identified 39 kinases likely to be involved in the phosphorylation signaling in PA. Among these, STE20-like serine/threonine-protein kinase (SLK) was statistically significant in the comparison between PA vs. non-PA patients, PA vs. HC patients in the LEV fraction, and PA vs. non-PA patients in the LEV fraction. SLK is involved in the cell cycle, being able to activate the membrane–actin linkers ezrin/radixin/moesin (ERMs), which plays a key role in the mitotic spindle-orientation guidance [52]. Among the statistically enriched kinases predicted by the KEA analysis, there was the serine/threonine kinase Casein kinase II subunit alpha (CSNK2A1), able to negatively regulate apoptosis [53], the tyrosine kinase nerve growth factor receptor (NTRK1), involved in cell survival, which can activate the MAPK cascade, and the Cyclin-dependent kinase 2 (CDK2), involved in the control of the cell cycle and the only one statistically overexpressed in PA samples. The tyrosine kinase Lyn, involved in tumorigenesis [54], was identified in the LEV, SEV PA versus non-PA, and in the overall PA versus non-PA samples, but not in the unprocessed total. Consistently, in a previous proteomic study on EVD CSF, we reported that Lyn, which best differentiated the MB samples from controls, was only detectable in the EVs, while it was undetectable in the total-CSF fraction. The two-dimensional GO enrichment analysis highlighted 60 significantly enriched GO annotation terms in the comparison between the PA and non-PA samples. Proteins associated with low-grade glioma, kinases, extracellular vesicle, MEROPS, cytoskeleton, and cell cycle proteins were enriched in the PA samples, consistent with the ability to metastasize. On the other hand, proteins associated with extracellular matrix, immunity, and endopeptidase inhibitor activity were enriched in the non-PA samples, i.e., downregulated in the PA. Notably, the downregulation of extracellular-matrix adhesion proteins in the PA samples is consistent with their pivotal role in the tumor microenvironment establishment and tumorigenesis [55]. CSF is the main component of the brain extracellular fluid (ECF), whose composition is similar [56], and a primary route for metastases [57]; therefore, CSF alterations relay great sensitivity to those linked to the tumor microenvironment. The volumes of waste CSF from the EVD allowed the isolation of CSF EVs. These can transport tumor-derived material, with a homing ability offering a non-invasive means for the identification of new biomarkers to monitor disease progression and treatment response [24,58]. EVs can regulate the tumor microenvironment and the communication between TME and its components [23]. SEVs are more enriched in proteins functioning as cell-to-cell messages, while LEVs relay the parent cell status [28]. As EVs have been shown to be able to cross the blood–brain barrier [59], their use is promising for liquid biopsy, as they can be isolated from the plasma.

The present results have potential implications for their translational value for clinical practice. Future studies can tell whether the PA biomarkers highlighted here can be detected with the same statistical efficiency in serum from patients, for non-invasive clinical detection and to assess the possibility of bypassing the invasiveness associated with CSF utilization, as recently proposed [60,61,62,63].

## 5. Conclusions

The current study provides valuable insights into the molecular landscape of childhood pilocytic astrocytoma (PA) through comprehensive proteomic and bioinformatic analyses of cerebrospinal fluid (CSF) obtained from extraventricular drainage (EVD), along with their extracellular vesicles (EVs), including both large and small EVs. EVs emerged as promising candidates for biomarker discovery in childhood PA, given their role in intercellular communication and transfer of bioactive molecules. Key findings include the identification of CPXM2 and AQP4 as proteins with high discriminatory potential in all three fractions. Furthermore, the CPXM2 protein was validated by ELISA in CSF EVD and by Western blot analysis in CSF EVD and biopsy tissue, demonstrating its potential as the most promising up-regulated biomarker for PA. It showed good diagnostic value in distinguishing PA from both non-tumoral controls and another brain tumor unrelated to PA. Additionally, gene ontology terms and the kinase profile associated with CSF EVD obtained from PA patients were elucidated, offering insights into potential therapeutic targets. These results highlight the translational potential of PA biomarkers identified in CSF EVD for non-invasive clinical detection, warranting further investigation into their utility in personalized medicine through liquid biopsy in blood and/or urine samples.

## Figures and Tables

**Table 1:** Clinical characteristics of all subjects present in the study. All patients with brain tumors have a histological diagnosis. Age is reported as years (median and range).

Groups	Discovery/Validation Cohort	Gender (M/F)	Age (Year)
Congenital hydrocephalus (CH)	8/18	14/12	1 (0–22)
Pilocytic astrocytoma (PA)	8/19	14/13	8 (3–15)
Medulloblastoma (MB)	8/13	11/10	5 (0–15)

## Data Availability

The original mass spectrometry data presented in the study is openly available in a public repository at https://www.ebi.ac.uk/pride/ with the following identifier: PXD050589.

## Supplementary Materials

The following supporting information can be downloaded at: https://www.mdpi.com/article/10.3390/cancers16061223/s1, Figure S1: Graphs of (a) large EVs (LEVs) and (b) size distribution of small EVs (SEVs) obtained from extraventricular drainage (EVD) of cerebrospinal fluid (CSF), assessed by dynamic light scattering. The graph shows a Gaussian distribution profile with an average peak at 1000 ± 70 nm or 100 ± 5 nm for LEVs or SEVs, respectively. No statistical differences were observed between SEV or LEV fractions obtained from CSF EVD of congenital hydrocephalus (CH), pilocytic astrocytoma (PA), or medulloblastoma (MB) samples. Figure S2: Venn diagram of all identified proteins. Venn diagram shows common and exclusive proteins identified. Numbers and circles represent the distinct proteins in each condition, respectively. Figure S3: Heatmap of ANOVA statistically significant proteins. Heatmap of 392 proteins identified using ANOVA test. In the heatmap, each row represents a protein, and each column corresponds to a group condition. Normalized Z-scores of protein abundance are depicted by a pseudo color scale, with red indicating positive expression, white indicating equal expression, and blue indicating negative expression, compared to each protein value, while the dendrogram displays the outcome of unsupervised hierarchical clustering analysis, placing similar protein-profile values near each other (see detail in Supplementary Table S1). Figure S4: Volcano plots of the comparison between pilocytic astrocytoma (PA) and congenital hydrocephalus (CH) patients, and PA and medulloblastoma (MB) patients in unprocessed (Tot), small- EV (SEV), and large-EV (LEV) fractions. Graphical representation of T-test analysis applied to the whole proteomic dataset. In the volcano plot, the x-axis and y-axis show the protein-profile variation between PA and CH (a, c, and e), and PA and MB (b, d, and f) in the Tot (a, b), SEV (c, d), and LEV (e, f) fraction, and their −Log10 *p*-value, respectively. The black curves show the statistical significance threshold. The plot shows the protein gene names of the most promising biomarkers in the discrimination of PA samples. Figure S5: Volcano plots of the comparison between pilocytic astrocytoma (PA) and non-PA patients, in unprocessed (Tot), small-EV (SEV), and large-EV (LEV) fractions, and between PA and non-PA patients, considering the three fractions together. Graphical representation of T-test analysis applied to the whole proteomic dataset. In the volcano plot, the x-axis and y-axis show the protein-profile variation between the PA and non-PA samples in the Tot (a), SEV (b), and LEV (c) fractions and between PA and non-PA patients, considering the three different fractions together (d) and their -Log10 *p*-value, respectively. The black curves show the statistical significance threshold. The plot shows the protein gene names of the most promising biomarkers in the discrimination of PA samples. Figure S6: Venn diagram of all statistically significant proteins. The Venn diagram shows common and exclusive statistically significant proteins identified by T-test analysis. Numbers and circles represent the distinct proteins in each comparison, respectively. Figure S7: Two-dimensional gene ontology enrichment analysis between pilocytic astrocytoma (PA) and non-PA samples. The two-dimensional scatter plot shows the enriched gene ontology annotation terms in the comparison between PA and non-PA in the unprocessed-total (square), small-EV (circle), and large-EV (triangle) samples. In the graph, the symbols located on the straight line passing through the coordinates (1x, 1y) and (−1x, −1y) are the equally enriched signatures, while those above or under this line are positively enriched in PA or non-PA samples, respectively. The red and blue symbols are the gene ontology annotation terms enriched above and below the 95% of CI, respectively. Figure S8: Bubble diagram of all kinases identified filtered for the 70% of identity in at least one condition. Bubble diagram of the 39 kinases identified by the MS approach. In the diagram, each row represents a kinase, and each column corresponds to a T-test comparison. Normalized Z-scores of Log_2-_fold change protein are depicted by a pseudo color scale, with red indicating positive expression, white indicating equal expression, and blue indicating negative expression, whereas the size was proportional to their −log_10_ *p*-value. Visual inspection of the diagram indicates the kinase enrichment in the small-EV (SEV) and large-EV (LEV) fractions compared to the unprocessed-total (Tot) fraction (see detail in Table S2). Figure S9: Western blot analysis. (a) Representative Western blot analysis of full-length gel (8–16T%) for CPXM2 protein in unprocessed-total fraction (Tot) of pooled CSF EVD obtained from medulloblastoma (MB) (lane 1), congenital hydrocephalus (CH) (lane 2), or pilocytic astrocytoma (PA) (lane 3) samples. The CPXM2 antibody detects a single band, corresponding to the predicted molecular weight of the entire protein. Furthermore, CPXM2 is more abundant in PA samples, compared to other CH and MB samples; (b) blue-silver staining of the same samples as in the previous panel is used as a loading control. Figure S10: Western blot analysis of paired cerebrospinal fluid (CSF) from extraventricular drainage (EVD) and biopsy tissue from patients with pilocytic astrocytoma. (a) Representative Western blot analysis of full-length gel (8–16T%) for CPXM2 proteins in CSF EVD (lanes 1, 2, and 3), and biopsy tissue (lanes 4, 5, and 6) from the same patients, with pilocytic astrocytoma. The CPXM2 antibody detects a single band corresponding to the expected molecular weight of the entire protein; (b) blue-silver staining of the same samples as in the previous panel is used as a loading control. Table S1: List of all identified proteins in the cerebrospinal fluid (CSF) of extraventricular drainage (EVD) in congenital hydrocephalus (CH), pilocytic astrocytoma (PA), and medulloblastoma (MB) patients for unprocessed-total (Tot), small-EV (SEV), and large-EV (LEV) fractions. The symbol “+” indicates the presence of protein. Table S2: List of all statistically significant proteins identified in the study. For each protein, we report the Log2 expression fold change, its −Log10 *p*-value after FDR correction for multiple interactions, and variable importance in the projection (VIP) score. The “+” symbol indicates statistical significance. Table S3: List of fifty proteins highlighted by the statistical analysis. The symbol “+” indicates the statistical significance of each protein in each comparison performed in the study. Table S4: Two-dimensional enrichment analysis between pilocytic astrocytoma (PA) and non-PA samples. List of all statistically significant Gene Ontology (GO) annotation terms enriched in the comparison of the PA and non-PA in unprocessed-total (square), small-EV (circle), and large-EV (triangle) samples. For each term, we report the enriched score, its *p*-value after FDR correction for multiple interaction, and GO type. The symbol “+” indicates the GO terms enriched above 95% of CI. Table S5: Kinase enrichment analysis. List of kinases potentially involved in the regulation of phosphorylation signaling in the comparison between pilocytic astrocytoma (PA) samples and non-PA samples. The enriched rank and protein substrate are reported for each predicted kinase.
