# Supplementary figures and images for: Proteomic Profiling of Cerebrospinal Fluid and Its Extracellular Vesicles from Extraventricular Drainage in Pediatric Pilocytic Astrocytoma, towards Precision Oncology
Source: Cancers (Basel). 2024 Mar 20;16(6):1223. doi: 10.3390/cancers16061223 (PMC10969024; doi:10.3390/cancers16061223)

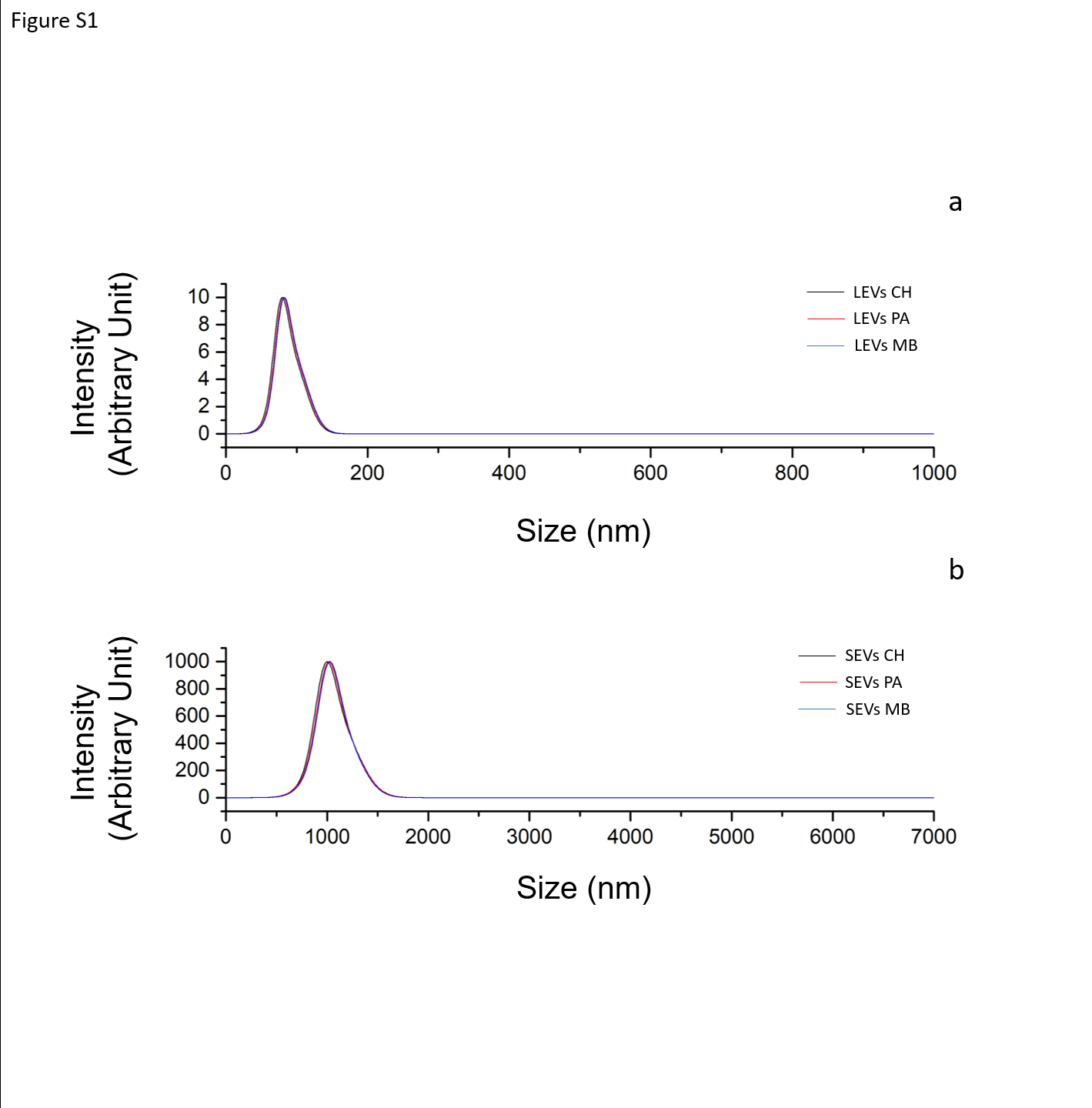

Supplement: Supplementary file 1 [file cancers-16-01223-s001.zip › Figure S1.jpg]

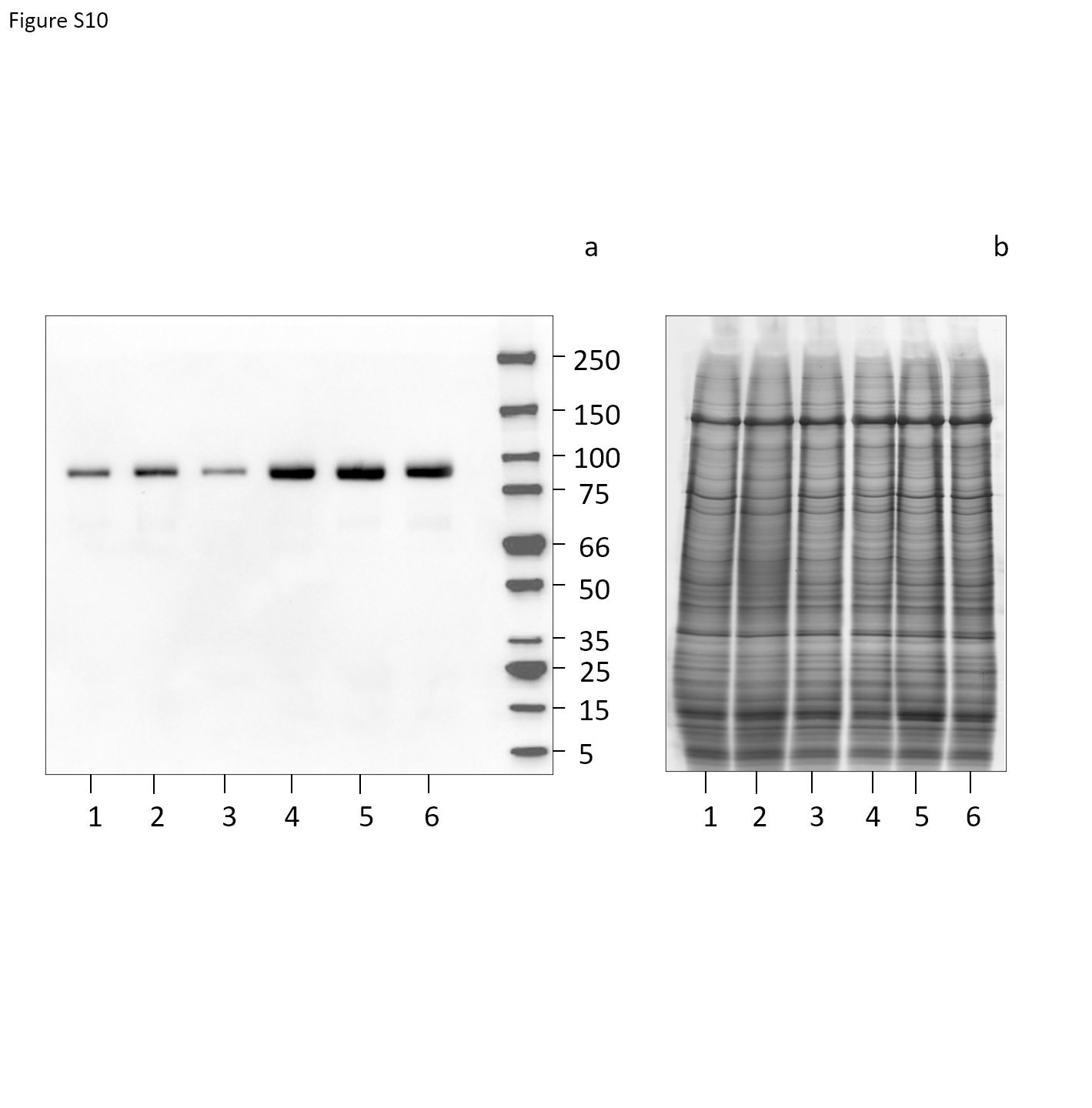

Supplement: Supplementary file 1 [file cancers-16-01223-s001.zip › Figure S10.jpg]

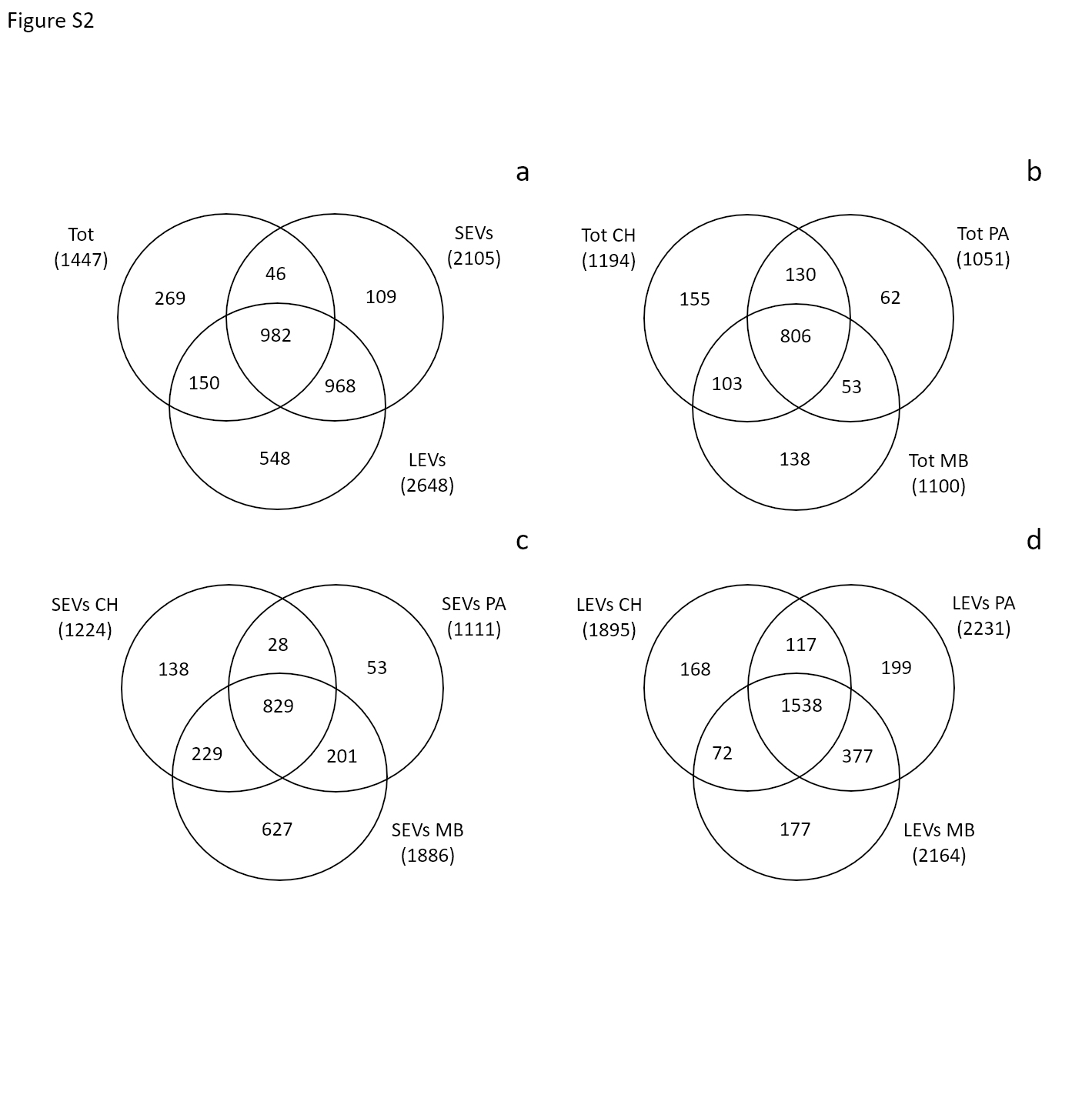

Supplement: Supplementary file 1 [file cancers-16-01223-s001.zip › Figure S2.jpg]

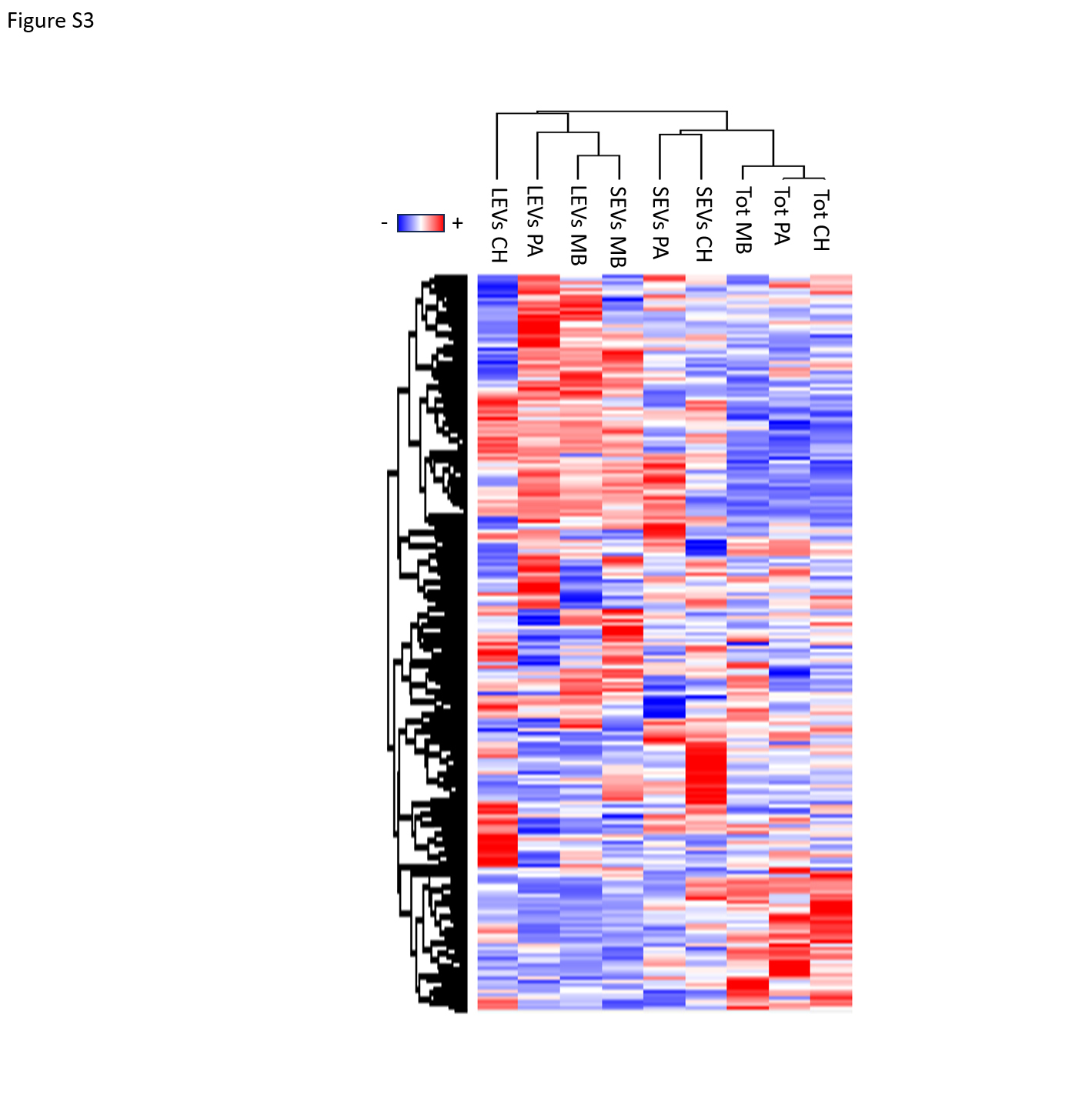

Supplement: Supplementary file 1 [file cancers-16-01223-s001.zip › Figure S3.jpg]

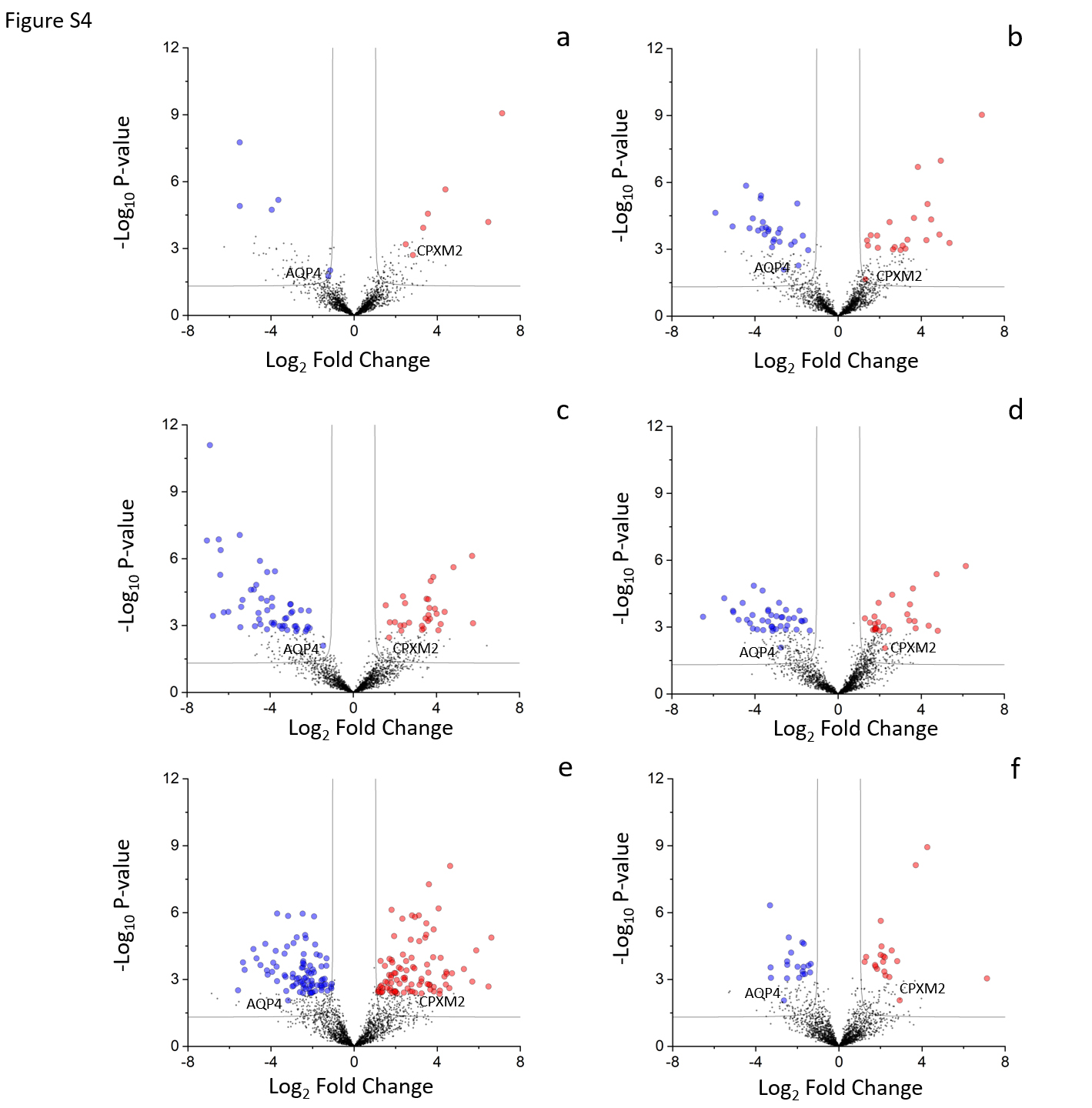

Supplement: Supplementary file 1 [file cancers-16-01223-s001.zip › Figure S4.jpg]

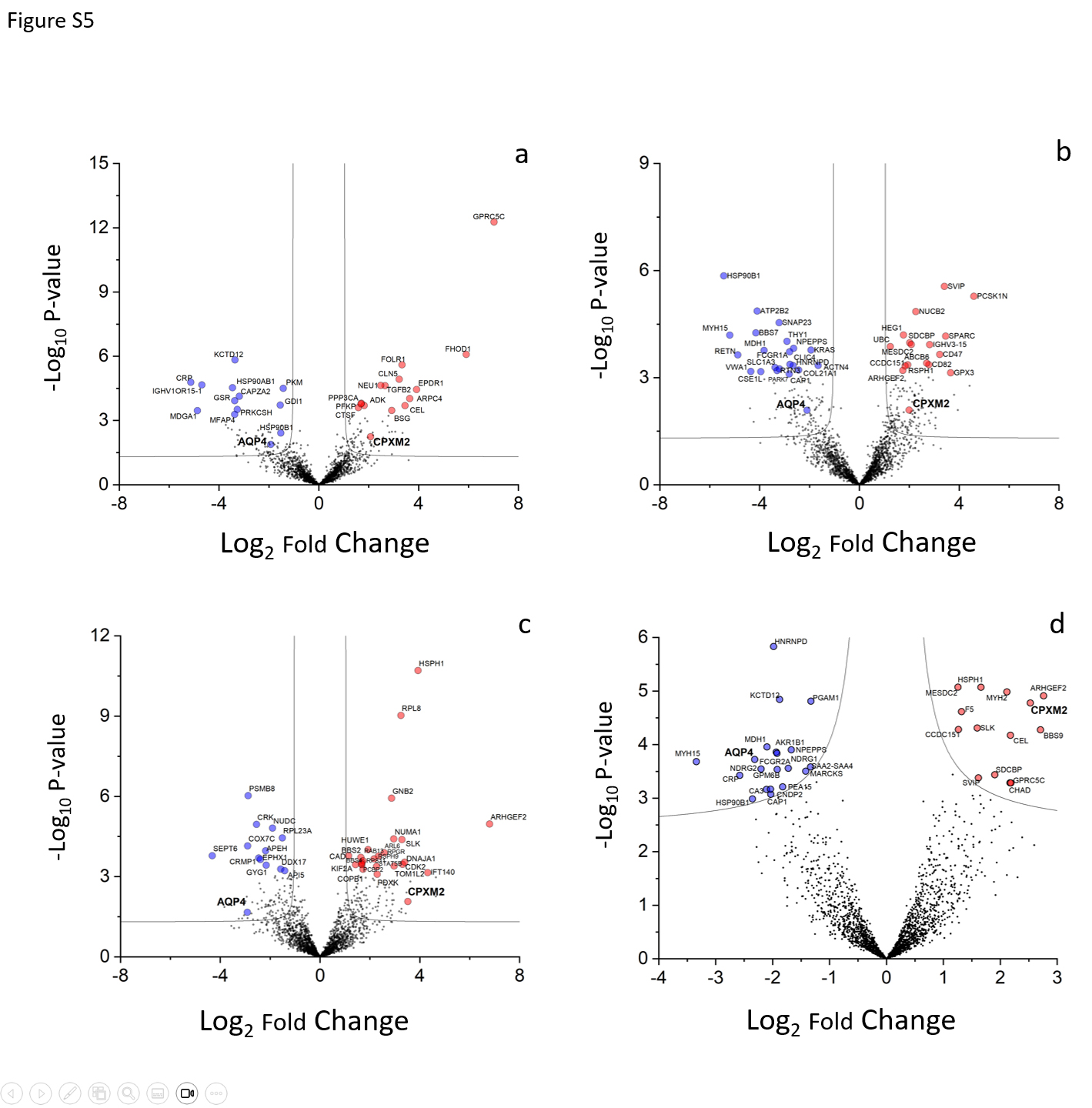

Supplement: Supplementary file 1 [file cancers-16-01223-s001.zip › Figure S5.jpg]

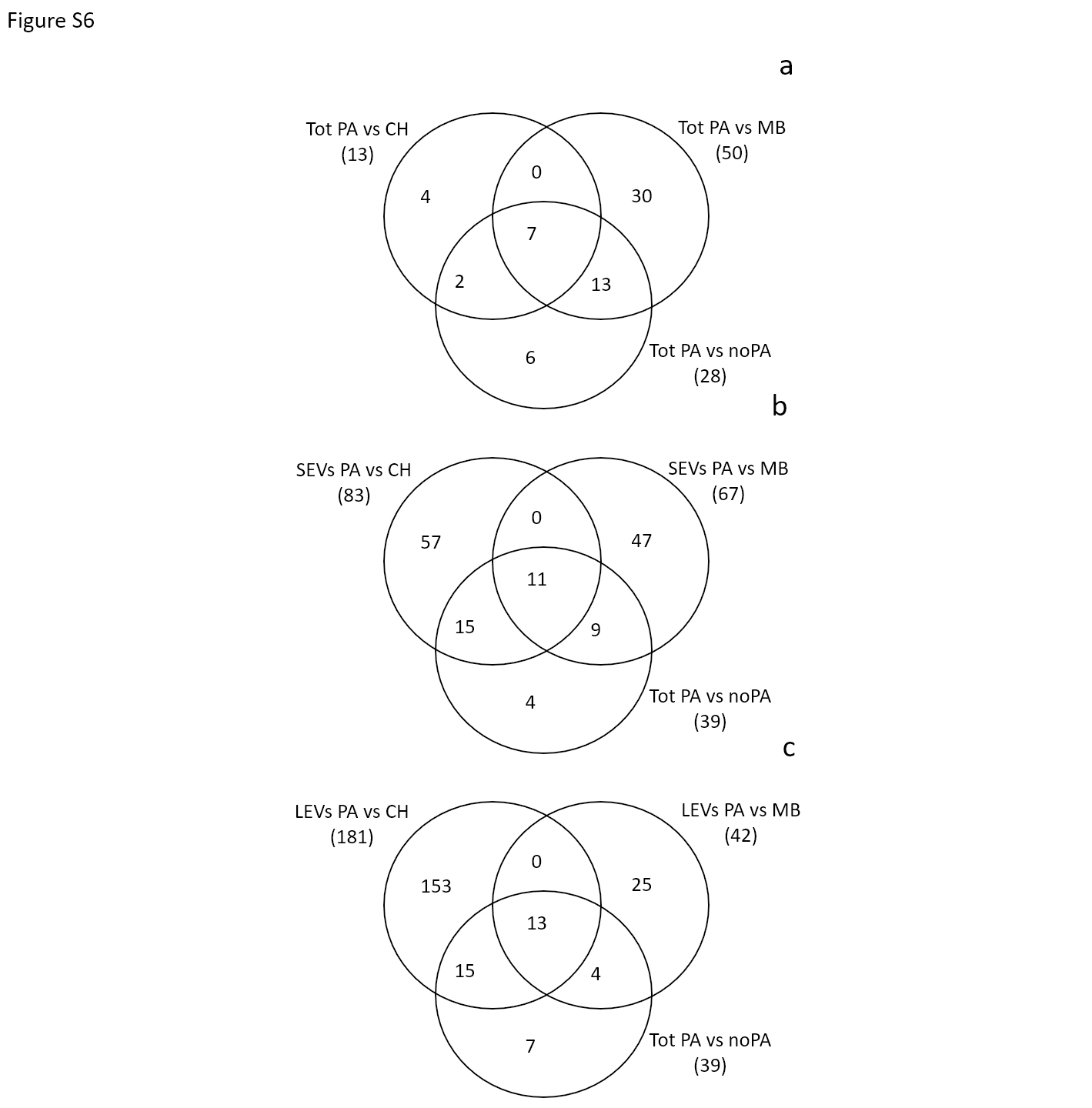

Supplement: Supplementary file 1 [file cancers-16-01223-s001.zip › Figure S6.jpg]

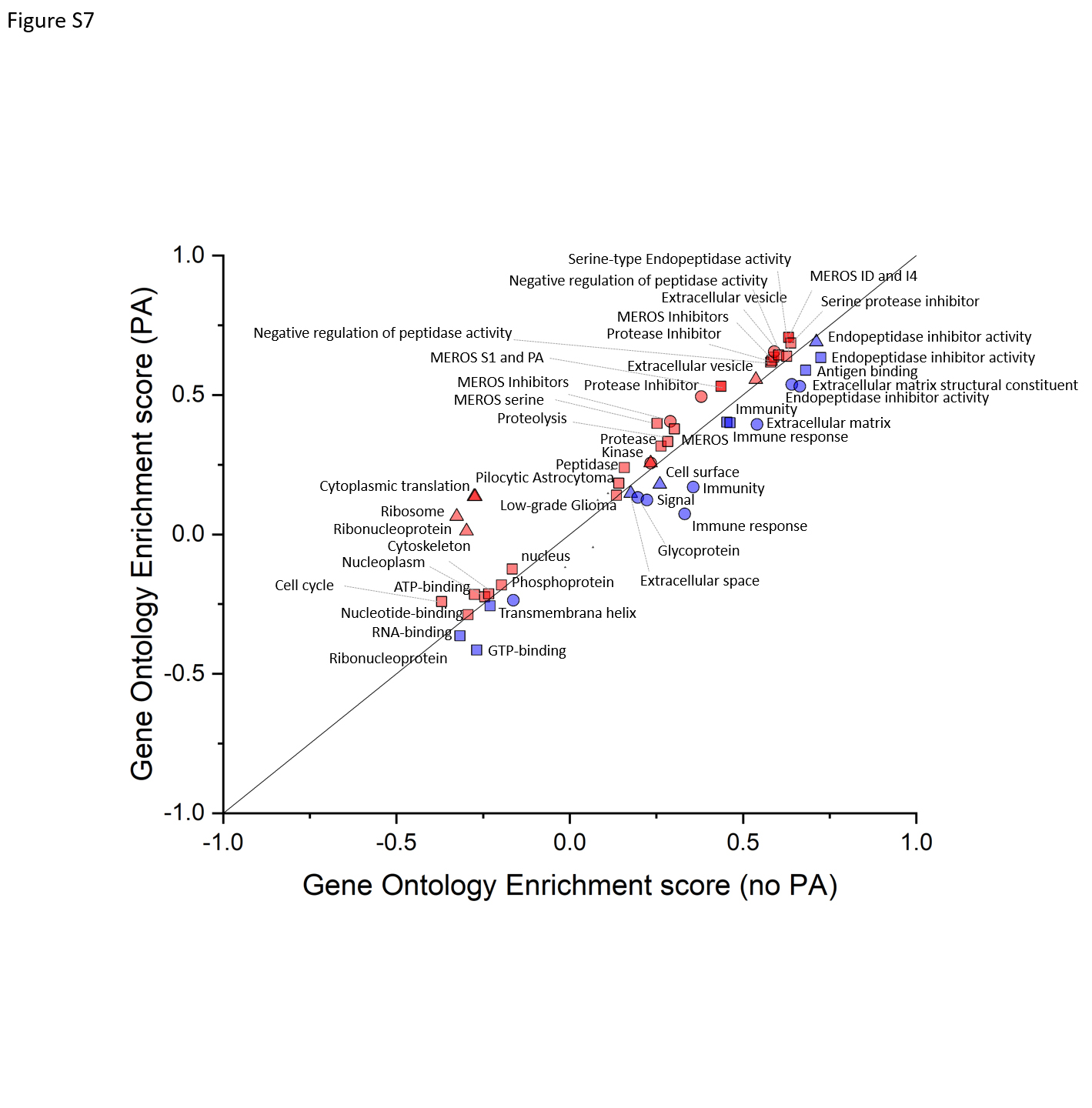

Supplement: Supplementary file 1 [file cancers-16-01223-s001.zip › Figure S7.jpg]

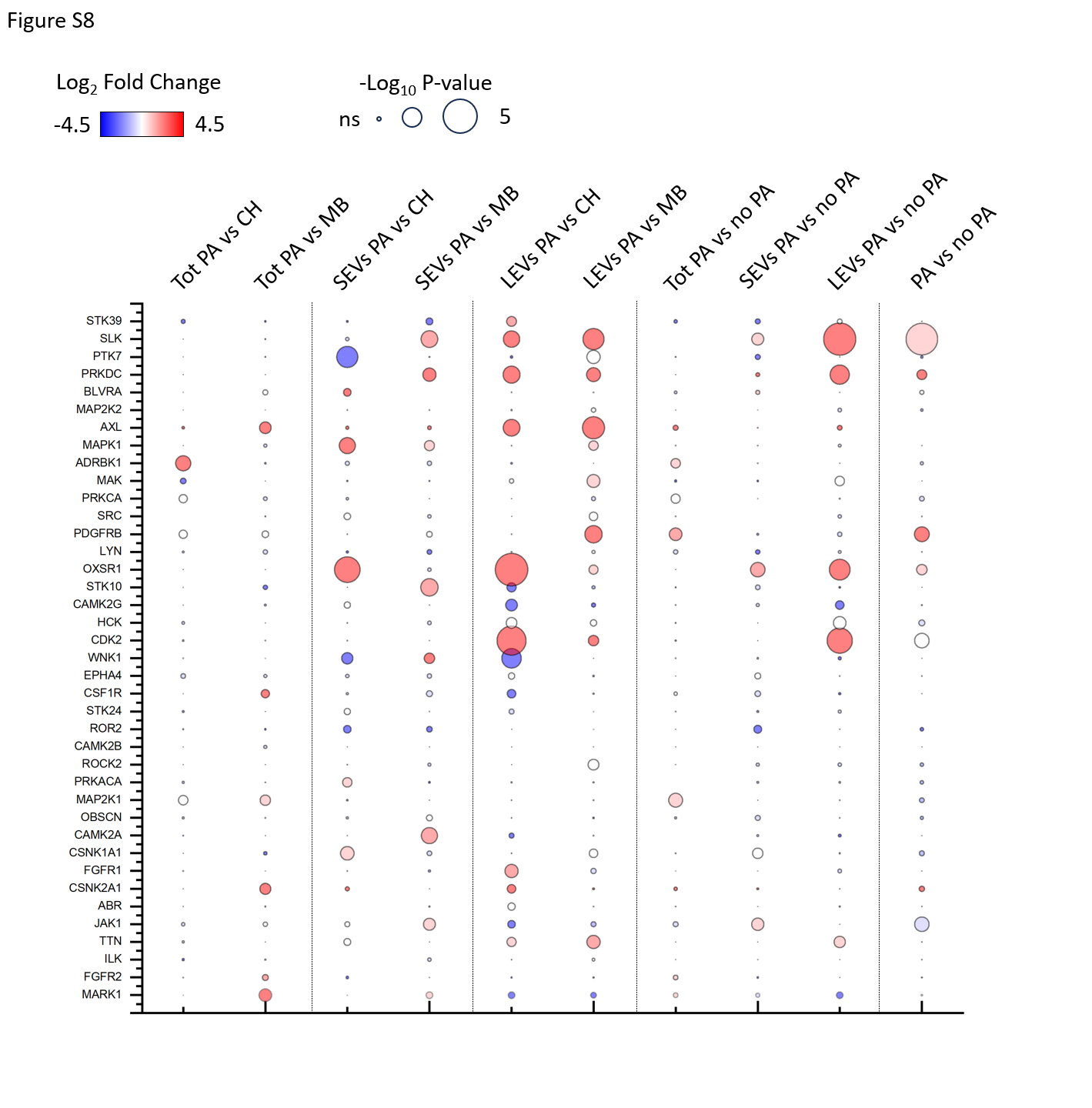

Supplement: Supplementary file 1 [file cancers-16-01223-s001.zip › Figure S8.jpg]

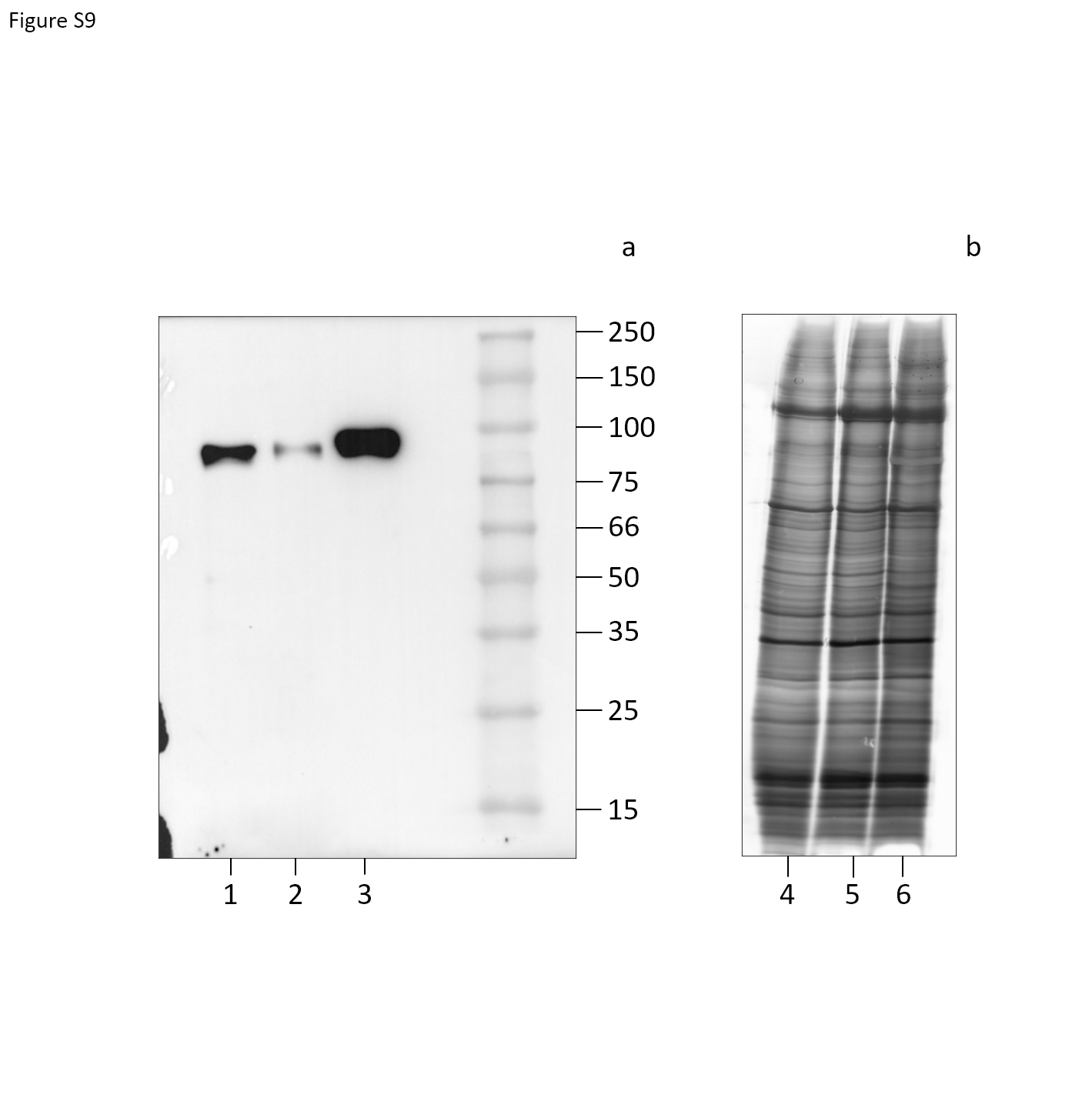

Supplement: Supplementary file 1 [file cancers-16-01223-s001.zip › Figure S9.jpg]
